# Because Others Do

**Published:** 1879-04

**Authors:** Ben. H. Pratt


### THE BISTOURY.

Vol. XV. ELMIRA, N. Y., APRIL, 1879. No. 1.

### BECAUSE OTHERS DO.

BEN. H. PRATT.

If every one of us could do just what we deem it right and proper to do, regardless of the acts or of the opinions of others, how many of us would live as we now live, talk as we now talk, dress as we now dress, eat as we now eat, spend as we now spend, do anything as we now do it ?

The idea lying topmost in the mind of the average human being about to undertake anything, whether important or not, whether in the way of business or pleasure, of work or recreation, is this: What will people—relatives, friends, neighbors, the circle in which he moves, the community in which he dwells—what will they think, say, or do, about it ? Every man has in mind somebody who represents, or seems to him to represent, the great public, and wonders how he or she will take the move he is about to make. He ponders the question over and over in his mind, and every ponderment increases the catalogue of phrases that this representative will use upon any attempt to carry out the project he has in mind.

And there is no one so self confident, so independent, so oblivious of this outside opinion or influence as to be himself and do that which he would. This dread of and shrinking from what may be said of us under any save the humdrum circumstances of life, is the great governing factor in the mind of each of us, entering into our life and becoming part and parcel of it. It is the bug-bear, the mole-hill-mountain, that presents barriers to our being and doing on every hand.

We may talk as loudly as we may, and as boldly as we may, about our independence, our utter disregard of public opinion, our determination to do that which we feel and know to be right, be the circumstances what they may, and the consequences what they may, and yet, when it comes right down to doing instead of planning, acting instead of thinking, putting into practice instead of theorizing, in ninety-nine cases out of a hundred, unless our planning, and thinking, and theorizing, jump with those of our friends, and are in a measure sustained by them, we shrink from even our plain duty in the matter, and yield up our project to their wishes, rather than face those fearful phantoms, the opinion, talk, looks and demeanor of those about us.

Custom, however ridiculous or compromising it may be, has made certain things doable provided we do them in the conventional way. It has made it not only perfectly proper and admissible, but even highly commendable to do some of the meanest things imaginable, provided we do these after the conventional formula, and as the dictum of what we call society, directs. What we may do does not seem to be so much a matter for consideration as how we shall do it. On the other hand, this same custom has as emphatically decreed that we may not, shall not, do even the most honorable and philanthropic deeds, unless we conform to a certain prescribed method in the doing thereof, as though we should apologize for obeying the dictates of our heart or conscience.

Because, forsooth, it has come down to us from a time whereof the memory of man runneth not to the contrary, that others have done certain things in a certain and formulative way, it has become the habit of people to think that these things can be done in no other way, and he who deviates by so much as an iota from that inherited plan, violates some of the laws which society has laid down for his guidance, and becomes at once a sort of social freebooter. Hence it is that men endeavoring to conform as completely as possible to the dicta of this social tyrant, become warped out of their natural selves and bent into a species of automata, which move only as they are directed by the strings and springs, the wires and wheels of the Grundy mechanism, which is the ill concealed motor of their social and business operations.

Men who have been ordinarily successful in life, and have acquired something more than a competence—who have surrounded themselves with all the comforts, and many of the luxuries of life—who live in something like what most are pleased to term, considerable style, and begin to be looked upon as men of influence and mark in a community because of such success and acquired position—let such, by some fortuitous chain of circumstances or the exigencies of business, lose their opportunities for continuing to so live and maintain their status in society—let such descend to a mode of living, which, though far inferior to their former style is nevertheless far from being a real deprivation, and one which need not be a bar to their happiness, it still entails a certain sort of misery, not by reason of circumscribed circumstances—not because they have not the means of being happy—not because they have not enough of the good things of life to meet their real needs—not because they have not still within themselves the means of enjoying what is left them—but simply because they are continually in dread, lest some one may be remarking upon their changed manner of living, and that those who knew them in their more prosperous days, may be drawing unpleasant comparisons between them, as then and now. Thus, for no other reason than they cannot do as they once did, and as others do with whom they have associated or still do associate, life is rendered a continued series of useless annoyances, and inexcusable wretchedness.

And not only are the once joyous homes made unhappy by this terror of what others think, but they, who under other circumstances might, nay, would set up other and new homes, are confessedly prevented from doing so by this same dread. Many of our young men of to-day will acknowledge their unwillingness to enter into the matrimonial state, because they cannot afford to support their wives in the same style they have become accustomed to as girls—cannot afford them all the luxuries that doting and well-to-do parents provide their daughters—cannot maintain the same position which the world, their little world, asserts they must continue to hold, or lose somewhat of that standing in society to which they are used, and young men, brave as they may be in every other matter of business, have not the courage to defy the opinion of others and live as well as they can—as well as, and perhaps better than their fathers and mothers lived when they set up homes for themselves. Our young men have become so strongly impressed with this notion of what society claims of them, that rather than do what they would, and what they know to be the right and honorable thing to be done, suffer bitter disappointments, and allow the best years of their lives to be sacrificed to the mere opinions of others.

And we are sorry to add that there is not in the young women of our time a sentiment antidotal to this, except in very rare instances—so rare that a case in which a daughter defies this opinion of the community is deemed sufficient to found a romance upon. Were such a sentiment less rare—were they who are wooed by young men of worth and intelligence, fearless enough to say that they could forego the opinion of the social circle in which they move, and would rather endure for a time the eye askance of meddlesome ones, than forfeit the love of an honorable man—were they content to sacrifice somewhat of their temporary luxuries in order to achieve the greater happiness in store for them, and thus show their contempt for a custom that would be more honored in the breach than in the observance—the frown of public opinion would soon become easily borne.

But even in the minor matters of talk and dress, we are influenced almost wholly by what is said and worn by others. There's a conventionalism of talk that when carefully scrutinized is quite as fruitful of difficulty, and as compromising as any of the other constraining influences. One may not in his conversation, overstep the circumscribed limits, without bringing down upon him that peculiar species of acrimonious criticism, which it is the province of small minds to indulge in. Does one occasionally allow himself to essay the philosophic, or the scientific, or the metaphysical, he becomes at once a target for the pop-guns of the superficial, and is dubbed a pedant. Superficiality, if not absolute vacuity of thought, is popular. That which can be rattled off, rather than thoughtfully spoken, is most kindly received for the reason that it is not *a la mode* to do anything strainful in the way of thinking. Mental effort, says society, is the province of the preacher, the professor, the lecturer. These are paid to manufacture ideas for those who want them, but as for the social, they prefer to drift hither and thither upon the tide of thoughtfulness, confining their conversation to that species of twaddle that comes of itself, requires no mental effort, scarcely demands even the attention of the hearer in order to be comprehended, and suggestive of nothing save more twaddle. This is "small talk," to make which is considered by many to be quite an accomplishment, and it is, if we regard the end attained as worthy of striving after. As an abstract art, perhaps it is deserving of especial admiration, very like rope-walking, standing on one's head, hanging by one's toes, juggling of all sorts and attainments of that ilk, which serve no definite purpose in life save to elicit wonder and arouse curiosity. It is well to do well whatever we strive to do, but as it would seem somewhat incongruous to devote one's self to any one of these accomplishments and expect to base one's claims to social eminence thereon, so is it absurd that one should base the same claims upon excelling in the art of making "small talk." Yet we do not overstate the matter, when we assert that the largest number of those known as brilliant members of society have no other accomplishment save this. It is the reigning power of the drawing room, and acknowledged as the supreme source of polite entertainments. It is the offspring of shallow mind, however, but having become popular, we have come to accept it as the custom against which it is not worth while to war. We must drift with the tide and surge with the crowd, as others do, because others do.

And he or she who defies custom in the matter of dress—who does not dress as others dress, however absurdly, however unbecomingly to his or her style of figure or face—becomes a social curiosity, whose peculiarities are bruited about upon the tongues of the conformists in a manner which would lead one to think a departure from the dicta of the modiste a crime unpardonable. Society will, in this particular, accept nothing but abject obedience to her queen, Fashion. No exemption is allowed for rich or poor, and worth of the highest order, unless it come clad in the conventional mode, is rejected because of its uncouth presentment. In no department of society is adherance to its commands so strictly enforced as in this. Whoso offends in this offends in all, and no offence is so acutely felt or so speedily punished by social ostracism as that of appearing in garments that have not upon them the stamp that fashion's latest whim has devised as the silent shibboleth of admission to "our set." It would seem the acme of the ridiculous to be governed by so silly an idea as this, had not social usage accustomed us to it.

In the matter of our every day food, society places its commands upon us, and but for the fact that the family board is not spread in the sight of the social inquisition, we might be compelled to eat according to the mandates of that uncompromising tyrant. That the individuals composing the body social, have one privilege left them, the right of domestic privacy for at least a few hours of each day, we feel truly grateful, since but for this we could not obey our gustatory sense, but must needs yield to the dictation of that self asserting autocracy, whose sway is only acknowledged when under its immediate control, and in presence of its official ignorami.

But it is in the matter of our expenditures, that the behests of custom must be most implicitly obeyed. In this particular, there is no opportunity to escape its penetration. Society enters your apartments, and inventories your effects with an eye to business far keener than that of the auctioneer or the sheriff. You are required to make verbal memoranda of the cost of your establishment, and of each article that goes to complete it, and present the same for inspection and approval to the self appointed agents of the social order, to which you belong. If the list is not long enough, or the sum total of cost not up to the prescribed standard, you are unhesitatingly informed as to what is expected of you, if you wish to remain a member in good standing, of this delightful organization. Your piano must be of the requisite make and value ; your paintings must be exhibited in sufficiently showy frames, which, to polite society, are the only criterion by which your art works in this line, are judged; your bronzes must bear the imprint—bogus or not, it matters not—of genuiness; your bric-a-brac must be likewise labeled; your servants must number sufficiently, to indicate a good degree of self-helplessness; your income must touch the point of independence; in fine, you must make a magnificient showing of pecunious indifference, and however shoddy or snobby, it all may be, you'll pass. Brains, intelligence, real worth, intellect—these are not required of you, for they are below par for want of a market.

Yes, we must live as others live, talk as they talk, dress as they dress, eat—well, we rather have it our own way as to eating—spend and do with singular exactness as they do.

Are we not fools? Verily we are of fools, the most degraded.

Coming—as the boy's say—right down to dots, what's the use of all this? How are we better for it? What is there in society to be dreaded in this way? What is society, anyway? Is it not but an aggregation of individuals? What is there to dread in an individual? One isn't so very much troubled if his neighbor Brown finds fault with him, or speaks slightingly of him, or avoids him, or indulges in compromising remarks concerning him. One merely says of Brown, that he don't amount to much, anyhow, for he's only Brown, after all. And Smith, suppose he gets his dignity crosswise, and says and does about the same thing of one? Why, he's only that man Smith, and why worry over what he may say or do? Now the whole social body is only an aggregation of Browns & Smiths, and unless one has really done a wrong thing, or violated his conscience in his conduct toward some of these Smiths or Browns, why allow the combined Smiths and Browns to worry one?

And after all, Society isn't half so terrible a creature as it is represented, providing we talk up to it—talk back to it—give it tit for tat—or ignore it by an indifference to its whims.

Society don't really, in its deep down heart, have any special ill will against people, but if it can lead you by the nose, and you stick up your nose to have it taken hold of, it will take advantage of the grip. It's a good deal according to how you act with society. You can bluff it off if you've got the spunk to do it. If you lapse from its requirements, or are by circumstances compelled to violate some of its commands, you may expect that its cordiality will be witheld—that your acquaintance will not be courted—that you will not be extended any favors—that it will let you pretty severely alone—but you may rest assured, that if you don't show yourself pettish and poutful under the treatment, you will be a good deal thought about, and your independence will rather redound to your credit. But here's the rub. They who have by some unfortunate—or fortunate—hook or crook, fallen out of the good graces of society, are apt to be rendered so exquisitely sensitive thereby, that they imagine everybody to be a species of enemy whom it were well to avoid. Avoidance begets a corresponding withdrawal on the other side, and an estrangement ensues which is almost universally the fault of him whom circumstances have embitered. The man who can keep up a semblance of familiar intercourse with those from whom some trick of fortune has separated him, will scarcely ever find himself rated much below the social level of those whose peer, financially, he once felt himself to be.

No, its only a bug-bear, this social ostracism which people are so fearful of, but so long as we feel that we must do as others do in order to be what others are, we are of slaves the most servile. Let us do the best we can, under our circumstances, but try to do no better, and we will find that it is not necessary to do as others do, in order to be what we ought to be.

